# Sleeping with the enemy: *Clostridium difficile* infection in the intensive care unit

**DOI:** 10.1186/s13054-017-1819-6

**Published:** 2017-10-22

**Authors:** Florian Prechter, Katrin Katzer, Michael Bauer, Andreas Stallmach

**Affiliations:** 1 Department of Internal Medicine IV, Jena University Hospital, Am Klinikum 1, 07743 Jena, Germany; 2 Department of Anesthesiology and Intensive Care Medicine, Jena University Hospital, Am Klinikum 1, 07743 Jena, Germany; 3 Center for Sepsis Control & Care, Jena University Hospital, Am Klinikum 1, 07743 Jena, Germany

**Keywords:** *Clostridium difficile* infection, Management, Intensive care, Critical care, Severe infection, Treatment failure, Antibiotic-associated diarrhea

## Abstract

Over the last years, there was an increase in the number and severity of *Clostridium difficile* infections (CDI) in all medical settings, including the intensive care unit (ICU). The current prevalence of CDI among ICU patients is estimated at 0.4–4% and has severe impact on morbidity and mortality. An estimated 10–20% of patients are colonized with *C. difficile* without showing signs of infection and spores can be found throughout ICUs. It is not yet possible to predict whether and when colonization will become infection. Figuratively speaking, our patients are sleeping with the enemy and we do not know when this enemy awakens.

Most patients developing CDI in the ICU show a mild to moderate disease course. Nevertheless, difficult-to-treat severe and complicated cases also occur. Treatment failure is particularly frequent in ICU patients due to comorbidities and the necessity of continued antibiotic treatment. This review will give an overview of current diagnostic, therapeutic, and prophylactic challenges and options with a special focus on the ICU patient.

First, we focus on diagnosis and prognosis of disease severity. This includes inconsistencies in the definition of disease severity as well as diagnostic problems. Proceeding from there, we discuss that while at first glance the choice of first-line treatment for CDI in the ICU is a simple matter guided by international guidelines, there are a number of specific problems and inconsistencies. We cover treatment in severe CDI, the problem of early recognition of treatment failure, and possible concepts of intensifying treatment. In conclusion, we mention methods for CDI prevention in the ICU.

## Background

*Clostridium difficile* infection (CDI) is a growing problem throughout the healthcare system both in hospitals and in preclinical settings. An analysis of US nationwide samples shows that the number of inpatients with CDI more than doubled from 2000 to 2010. The number of CDI-associated megacolon cases almost tripled, and the mortality rate almost doubled [1]. Total deaths associated with CDI in the USA in 2011 were estimated at 29,000 [2]. The Center for Disease Control and Prevention classified CDI as an urgent threat and estimated that up to US$3.8 billion in medical costs could be saved over 5 years by implementing adequate preventative measures.

CDI has a particular impact on patients in intensive care units (ICUs). Most authors report a prolonged length of stay in the ICU [3, 4] as well as higher ICU costs [5] and higher mortality rates [6] for CDI patients. Besides this, the current practice of isolation poses significant logistic and economic challenges.

## Prevalence and severity of CDI in the ICU

Among ICU patients, diarrhea is one of the most common symptoms. About 15–38% of patients develop at least one episode of diarrhea [7–9]. In most cases, the cause of diarrhea is noninfectious and associated with complications of enteral feeding. According to data from North America and Europe, 11–13.5% [4, 7] of patients with diarrhea are diagnosed with CDI, leading to an estimated total prevalence of CDI in ICU patients of about 1–2% [4] with an incidence of 8.7 [10] to 53.9 [3] cases per 10,000 patient days.

The spectrum of disease ranges from relatively benign to highly complicated and potentially lethal. The severity of disease is defined by a range of clinical parameters (Table 1). Estimating the probable clinical course is essential for initial therapeutic decisions. According to a study by Bouza et al. [10], 28.6% of CDI cases among unselected ICU patients in a large Spanish teaching hospital are severe. The authors’ own, unpublished data indicate that only 12% of patients with CDI on our medical and surgical ICU meet the IDSA criteria for severe CDI.

**Table 1:** Guideline definitions for CDI severity

Severity	Infectious Diseases Society of America	European Society of Clinical Microbiology and Infectious Diseases	American College of Gastroenterology
Mild disease			Diarrhea as only symptom
Moderate disease			Symptoms apart from diarrhea not meeting the definition of severe or complicated CDI
Severe disease		Serum albumin < 30 g/l or	Serum albumin < 30 g/l and
	Leukocytosis > 15,000/µlor	Leukocytosis > 15,000/μl or	Leukocytosis > 15,000/μl or
	Creatinine > 1,5 × ULN	Creatinine > 1.5 × ULN or	
		‘Clinical markers of severe colitis’ (i.e., fever, rigors, shock, respiratory failure, peritonitis, ascites, ileus, elevated serum lactate, pseudomembranes)	Abdominal Tenderness
Complicated disease	Hypotension / shock or	Significant systemic toxin effects and shock with need for ICU admission, colectomy, or death	Admission to ICU, hypotension, fever > 38.5 °C, mental status changes, ileus or significant abdominal distension, serum lactate > 2.2 mmol/l, leukocytosis > 35,000/μl, signs of end-stage organ failure
	Ileus, megacolon		
Fulminant CDI	Not defined	Not defined	Not defined

*CDI Clostridium difficile* infection, *ICU* intensive care unit, *ULN* upper limit of normal

Stratification of patients into those with mild, moderate, severe, or severe and complicated disease is not consistent throughout the different guidelines (Table 1). On this subject, Kahnafer et al. [11] found rates of severe CDI differing between 11.6 and 59.2% just by applying different definitions to the same patients.

The main difficulty in finding a universally accepted classification for disease severity consists of determining a set of clinical parameters which can correctly predict the course and prognosis of CDI for patients in different clinical settings. A number of studies have attempted to identify factors that can reliably predict unfavorable outcomes (Table 2). The authors’ own data suggest that CRP, hypotension as well as an early decline in renal function are independent markers for increased mortality.

**Table 2:** Synoptic overview of suggested markers to predict disease severity in CDI

Prediction markers
Declining renal function [14] Treatment with systemic antibiotics [13] Age [12–14, 79] White blood cell count [13, 79] Albumin [79] Steroid therapy [79] Admission due to diarrhea [12] Recent abdominal surgery [12] Hypotension [12] Diagnosis of CDI in the ICU [12] Gender Immunodeficiency Readmission with recurring infection Transfer to ICU because of CDI Emergency surgery Ribotype O27 Lactoferrin, fecal calprotectin
No predictive value
Fever [79] Hemoglobin [79] Intensity [79] Comorbidity (diabetes, cancer) [79]

*CDI Clostridium difficile* infection, *ICU* intensive care unit

Hensgens et al. [12] proposed a prediction model involving age, admission due to diarrhea, recent abdominal surgery, and hypotension. Another reported scoring system is the ATLAS score involving age, treatment with systemic antibiotics, leukocyte count, albumin, and creatinine [13], or the CPR tool including age, serum creatinine, and leukocyte count [14]. Khanafer et al. [11] found that, throughout different definitions of severe CID, serum albumin and the presence of renal disease are consistent markers for a probable poor prognosis.

**Fig. 1:**
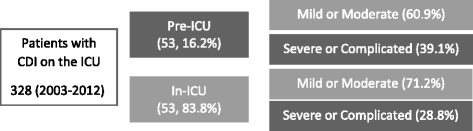
Mild or moderate versus severe cases of CDI depending on the primary point of diagnosis. *CDI Clostridium difficile* infection, *ICU* intensive care unit (Adapted from [10])

The aforementioned report by Bouza et al. [10] confirms that it is reasonable to expect a more severe or complicated course of CDI if the patient has been transferred to the ICU after the initial diagnosis (Fig. 1).

There is evidence that patients with a higher Sequential Organ Failure Assessment (SOFA) score at the time of diagnosis also have a higher risk of ICU mortality or severe complications [15].

In conclusion, out of the 2% of ICU patients with CDI, a significant number of cases can be classified mild or moderate. As there are no disease-specific markers, severity is measured by general parameters (e.g., leukocytes, renal function) frequently altered in ICU patients either because of the CDI or because of the patient’s underlying condition. None of the aforementioned parameters or scoring systems has been validated in the subgroup of ICD patients. It is difficult to separate the otherwise critically ill patient with mild CDI from the patient who is critically ill because of severe CDI. Current classification according to the international guidelines probably does not accurately reflect the actual risk profile of the ICU patient.

## Diagnosis

There is common agreement that testing for CDI should be part of the diagnostic routine in patients with diarrhea, defined as more than three loose stools within 24 hours [16]. According to all guidelines, testing should primarily be reserved for symptomatic patients [17]. Nevertheless, particularly in the ICU and in patients with severe CDI, there are cases where the classical symptom of diarrhea is replaced by intestinal paralysis or toxic megacolon. While there is no systematic analysis concerning patients with atypical presentation, numerous case reports can be found in the current literature (e.g., [18]). Consequently, it may be advisable to test for CDI in selected cases of nondiarrheic patients if there is high clinical probability of CDI. This can be done using stool samples or rectal swabs [19].

The clinical suspicion is mainly determined by a compatible clinical picture (diarrhea, abdominal sepsis with fever and leukocytosis, ileus, or toxic megacolon) in the absence of an alternate explanation and the presence of individual risk factors (e.g., previous antibiotic therapy, age > 65, hospital stay within the last 3 months, previous CDI).

There are various approaches to diagnosing CDI. Those include culture, molecular testing (nucleic acid amplification test, NAAT or PCR) for the gene encoding toxin B, toxin testing via enzyme-linked immunosorbent assay (ELISA), and antigen testing (e.g., testing for glutamate dehydrogenase (GDH)). Stool cultures followed by identification of a toxigenic isolate are considered the diagnostic gold standard.

Particularly in severely ill patients, the time needed for stool cultures may cause an intolerable delay in initiating therapy or further diagnostic procedures. Point-of-care testing (POCT) in the ICU using NAAT has been investigated [20] and could become more widely available in the future. At the same time, all diagnostic methods using NAAT (POCT, stool testing in the laboratory, or rectal swabs) can result in significant overdiagnosis of CDI [21]. In this respect, it is important to be aware of the fact that approximately 10–20% of all patients are colonized with toxigenic and nontoxigenic strains of *Clostridium difficile* without showing clinical symptoms. A screening study in ICU patients showed that while about 10% of a total number of 922 patients tested positive for *C. difficile* by NAAT, only about 3% developed a symptomatic disease [22]. Recent data confirmed that 11.8% of asymptomatic patients and 15.4% of symptomatic patients are found positive for *C. difficile* using a NAAT test [23].

This is supported by the observation that not all patients with positive polymerase chain reaction (PCR) results also have detectable toxin levels. The presence of toxin levels has been shown to be critical for patients’ prognosis [24]. It is not clear what consequences an asymptomatic colonization with *C. difficile* has for the patient; it may either pose a risk for developing CDI [25] or be protective against progression to symptomatic CDI [26]. Also unknown is whether a symptomatic patient with positive NAAT and negative toxin testing benefits from treatment for CDI. Because of this lack of evidence, the current practice of extensive testing and isolation has to be put into question. Positive NAAT alone cannot be taken as a reliable indication for treatment.

There are ambiguous statements in the current guidelines concerning a one-step or two-step diagnostic model in which a relatively nonspecific antigen test is followed by a specific confirmation test. The latest European guidelines highly recommend any two-step algorithm which includes the toxin test (by ELISA) to confirm the diagnosis [27]. There have been reports of undiagnosed cases of *C. difficile* in spite of stool testing due to nonsensitive diagnostic tests or lack of request [28]. In some countries, this is addressed by routinely sending microbiological samples to a central reference laboratory even if there is low clinical suspicion of CDI (younger patients, community-acquired diarrhea).

After microbiological confirmation, it is possible to determine the *C. difficile* ribotype. There are certain “hypervirulent” strains, notably O27, which are associated with a higher production of toxin, more cases of recurrent CDIF, as well as a more severe course of disease.

Endoscopy may prove helpful because it can deliver a rapid diagnosis if pseudomembranes are visualized. In addition, the presence of pseudomembranes is an ominous marker in itself for the course of the disease [29]. The chance of an immediate result has to be balanced with the potential risk of perforation, particularly if there is a high degree of inflammation as well as the low sensitivity. There is evidence that about 25% of CDI patients can be diagnosed based on imaging or endoscopic findings [4]. We generally limit endoscopy to cases where biopsy is necessary to exclude alternative diagnoses.

## Treatment

### Mild or moderate disease

According to international guidelines, patients with mild or moderate disease should be treated with oral vancomycin (125 mg qid) or metronidazole (500 mg tid) [16, 17, 30]. While the strain type is not taken into account in those recommendations, we recommend treating every infection with a known hypervirulent strain (particularly O27) as severe disease.

In the report by Bouza et al. [10], 75% of all CDI patients in the ICU were treated with metronidazole as monotherapy. If oral treatment is not possible either because of the patient developing toxic megacolon and ileus or because of a general impairment in intestinal motility shown to occur in a significant number of ICU patients [31], metronidazole can be applied intravenously. In our eyes, there are numerous disadvantages of this approach. For one, there are reports of decreased susceptibility [32] and an increasing rate of clinical failure with metronidazole [33, 34]. While there are currently no established mechanisms of resistance, some strains may respond to subinhibitory drug concentrations by forming biofilms [35]. Fecal drug levels are low even when applied orally. Intravenous application results in highly variable levels dependent on the patient’s stool consistency [36]. In addition, there have been reports of encephalopathy after prolonged metronidazole treatment of CDI [37]. In our opinion, All of this taken together with a faster time to resolution of clinical symptoms, higher success rates, and lower mortality rates for treatment with vancomycin [38] indicates that, particularly in the ICU, metronidazole can at best be considered second-line therapy. In critically ill patients with mild CDI, we favor treatment with oral vancomycin. If this is not feasible, alternatives include topical vancomycin (i.e., by enema or endoscopic catheter) or intravenous tigecycline. Those options will be discussed in the following paragraphs.

### Severe or complicated disease

First-line treatment of severe or complicated cases consists of oral vancomycin. While cure rates for vancomycin were estimated at around 78.5% for severe CDI in a randomized multicenter study [39], there are currently no reliable data for ICU patients.

The intravenous form of vancomycin can be applied orally, by nasogastric tube, or topically in the case of ileus or gastrointestinal discontinuity (e.g., ileostomy). There have been multiple, mainly retrospective, reports of successful topical treatment with vancomycin (500–1000 mg qid) by enema [40] or by endoscopic catheter placement in patients with megacolon [41]. There is currently no study directly comparing these forms of application to the oral application of vancomycin. Furthermore, a few more recent studies could not show a benefit of topical vancomycin as adjunctive therapy [42, 43]. As a side note, there is evidence that the application of oral or topical vancomycin in CDI patients can result in measurable serum vancomycin levels (>2.5 μg/ml), sometimes reaching therapeutic levels [44]. The consequences of this, particularly with regard to nephrotoxicity as well as development of vancomycin resistance, are not clear.

Fidaxomicin is a relatively new alternative to vancomycin. This very specific antibiotic is considered far less detrimental to the normal colonic microbiome than vancomycin [45]. There is evidence of equal treatment outcomes and lower recurrence rates compared with vancomycin in a collective of unselected CDI patients [46], and there are case series hinting that these benefits may also hold true for critically ill patients [47]. Fidaxomicin is recommended for the treatment of severe CDI in the guidelines, even though noninferiority in those patients still remains to be proven.

Recently, primary treatment of severe CDI with tigecycline has been reported to be superior to the standard treatment with vancomycin in a large retrospective cohort study [48].

### Treatment failure

Insufficient response to the aforementioned first-line treatments or “treatment failure” is considered one of the most serious problems associated with CDI in the ICU. One clinical problem yet to be resolved is the early identification of unresponsive patients to enable the timely escalation of treatment. In studies, treatment response is generally defined as < 3 loose bowel movements a day for more than 2 consecutive days. This definition is of limited use in the ICU where patients may have diarrhea from multiple causes (e.g., enteral feeding). Stool testing as proof of cure is strongly advised against in all guidelines. There are case reports in which procalcitonin was observed to be associated with the clinical course [49]. Overall, there is an unmet clinical need for reliable follow-up parameters.

Once it has been established that the patient is not sufficiently responding to first-line medication, there is the question of what the next therapeutic step should be. Although there are a number of possible treatments, none of those have been examined versus vancomycin as first-line therapy. The currently available data are unfortunately still mostly limited to case reports and case series.

Like vancomycin, oral teicoplanin is a long-established glycopeptide antibiotic. Oral teicoplanin was shown to be noninferior to vancomycin in an unselected group of patients [50]. More recent data indicate that it may be superior to vancomycin in efficacy and safety [51]. There have been reports of cure for ICU patients with severe CDI unresponsive to a standard scheme of oral and intracolonic vancomycin and intravenous metronidazole [52]. Unfortunately, there is only low evidence to support the use of teicoplanin even though it is the cheaper and older drug.

Although there are still no randomized studies on the combination of oral vancomycin and intravenous metronidazole, a recent retrospective analysis showed improved mortality rates (15.9% vs 36.4%) in critically ill patients treated with this combination [53]. There has been criticism concerning the statistical methods applied in this study as well as the small number of cases. Furthermore, animal experiments did not show a benefit for the combination treatment compared with vancomycin alone [54].

There are a growing number of reports of successful treatment of ICU patients with tigecycline [55]. Britt et al. [56] reported cure rates in four of five patients with a triple combination therapy comprising tigecycline, vancomycin, and metronidazole. Disappointingly, a more recent study showed no benefit for tigecycline as adjunctive therapy. However, it has to be pointed out that this study was performed with an unconventional combination of oral vancomycin, intravenous metronidazole, and tigecycline as primary therapy, a relatively small and unbalanced number of patients (90 patients, 21 treated with tigecycline), and a retrospective, nonblinded design [57].

Various other antibiotics such as rifaximin, surotomycin, cadazolid, tolevamer, ramoplanin, and fusidic acid have been reported to be effective against *C. difficile* either in vitro or in single case studies. There are no current data regarding their effectiveness in severely ill patients.

Another therapeutic approach to CDI has been the attempt to attenuate the effects of the toxins by application of intravenous immunoglobulin or specific antibodies (bezlotoxumab and actoxumab). While intravenous immunoglobulin has been employed in critically ill CDI patients with limited success [58], the antibodies have primarily shown a decrease in CDI recurrence rates but have not proven efficacious in the primary treatment of severe CDI.

Fecal microbiota transplantation (FMT) has been employed in recurring or relapsing CDI showing cure rates of about 90%. Meanwhile, there are first reports on the use of FMT as salvage therapy in the treatment of patients with severe CDI following treatment failure (Table 3). Unfortunately, most studies do not include detailed information regarding the number of patients treated in the ICU, and there has never been a comparison between FMT and current first-line treatment schemes. Thus, FMT can currently not be recommended as first-line treatment for severe CDI in the ICU setting. This is particularly regrettable as there are hints that earlier FMT may improve the effectiveness of the treatment [59].

**Table 3:** Studies on primary FMT in nonrecurring CDI

Study group	Patients included	ICU patients	Cure rates	Description
Agraval et al., 2015 [80]	146 total (57 with severe or complicated CDI)	?	82.9% primary 95.9% overall	All patients 65 years and older Varying protocol for FMT
Aroniadis et al., 2015 [81]	17 with severe or complicated CDI	?	88.2% primary 94.1% overall	
Fisher et al., 2015 [82]	29	13	62% primary 93% overall	Treatment protocol with FMT (colonoscopy) and continued vancomycin
Pecere et al., 2015 [83]	1			Altered protocol with FMT and fidaxomicin
Zainah et al., 2014 [84]	14 with severe CDI	6	79%	FMT via NGT
Lagier et al., 2015 [59]	61 patients with CDI (O27)	?	81.25% (vs 35.6%)	42 patients treated with antibiotics 3 patients treated with “tardive” FMT 16 patients treated with antibiotics and early FMT
Kelly et al., 2014 [85]	80 total (34% severe CDI)	?	78% primary 89% overall	Study on immunocompromised patients; disease flares in 14% of patients with inflammatory bowel disease
Gweon et al., 2016 [86]	7 total (2 with severe CDI)	?	100% overall	Study on older, multimorbid patients with primary FMT (administered orally)

*CDI Clostridium difficile* infection, *NGT* nasogastric tube, *FMT* fecal matter transplantation

Despite all these options, there will still be a percentage of patients in whom the clinical situation deteriorates and surgery becomes inevitable. It is an accepted fact that, if unavoidable, surgery should take place as soon as possible. Here again we are confronted with a lack of early, direct markers of disease progression. Serum lactate levels and white blood cell count are often employed as surrogate parameters. There has been a recent positive report on the scoring system by the University of Pittsburgh Medical Center (UPMC) for this purpose [60] (Table 4).

**Table 4:** UPMC scoring system for *Clostridium difficile* severity

Criterion	Points
Low albumin	1
Fever	1
Admission to ICU	1
Chronic medical condition	1
Pancolitis, ascites, and/or bowel wall thickening in CT scan	2
Elevated white blood cell count	2
Increased creatinine	2
Clinical signs of peritonitis	3
Hypotension requiring vasopressors	5
Respiratory failure due to *C. difficile*	5
Mental alterations	5

Total: 1–3, mild to moderate disease; 4–6, severe disease; 7 or more, severe complicated disease; 15 or more, high probability (75%) of treatment failure and need for surgery (Adopted from [60])

*UPMC* University of Pittsburgh Medical Center, *CT* ﻿Co﻿mputed ﻿﻿Tomography

According to older data, 1.1% of CDI patients and 29.9% of patients with severe CDI traditionally underwent surgery. Most often (90%), surgeons had to perform subtotal colectomy with ileostomy. Because of the preoperative comorbidity of patients, 30-day mortality rates were at a devastating 41.3% [61]. A study on minimally invasive ileal diversion with colonic lavage (ClinicalTrials.gov NCT01441271) referred to in the European guidelines was terminated due to a marked decrease in the number of eligible patients. It may be hoped that this is the result of the increasing effectiveness of medical treatment.

## Prevention

A recent systematic review suggests that the ICU setting is associated with a higher prevalence of new CDI, resulting in increased mortality [6]. In this context, we will discuss the issues of improved screening for patients at risk for CDI, optimization of antibiotic therapies, the issue of transmission of CDI within the ICU, and novel pharmaceutical approaches to reduce CDI rates.

### Screening for risk factors

Screening for individual risk factors and implementing an adequate intervention strategy can significantly reduce CDI rates [62]. In a model study, all patients aged 55 years or older with a hospital length of stay > 5 days were screened for their specific risk constellation. All patients considered at risk (i.e., history of CDI, immunosuppression, therapy with more than three antibiotics, prolonged mechanical ventilation, enteral feeding, low serum albumin) received a bundle of preventive measures (optimized hygiene, reevaluation of medication particularly concerning antibiotics and proton pump inhibitors (PPIs), probiotics). As a result, the incidence rate of CDI was significantly lowered. A multitude of additional risk factors for CDI have been mentioned in various reports. Generally, almost everything adding to the morbidity of patients also increases their risk profile. A recent meta-analysis reported age, admission in the past 60 days, mechanical ventilation, dialysis, history of congestive heart failure, and history of antibiotic treatment as the main risk factors for developing primary CDI [63]. While there are contradictory data on the correlation of PPI medication and CDI, PPIs were found to be an independent risk factor for the development of CDI in a study performed specifically in ICU patients [64]. Combined with evidence that prolonged use of PPIs reduces microbial diversity and thus increases susceptibility for CDI [65], we consider medication with PPI another major risk factor.

### Antibiotic stewardship

The leading cause of CDI is antibiotic treatment, most likely leading to severe alterations in the intestinal microbiome. There have been multiple studies showing that antibiotic stewardship (ABS) programs can reduce CDI rates [66]. The main targets for modifying antibiotic prescription habits are often the prophylactic antibiotic schemes. Even though there have been reports of alternative preoperative schemes less likely to cause CDI [67], the main issue is to make sure that preoperative antibiotics are not routinely continued after surgery.

Another target should be to reduce the prescription of antibiotics with a known association to higher CDI rates. The substances mainly talked of in that respect are the “4C” (clindamycin, cephalosporins, co-amoxiclav, and ciprofloxacin). In spite of this, even though certain antibiotics are more prone to triggering CDI, no antibiotic can be considered “safe” in this respect. Particularly, the combination of high-risk antibiotics and PPIs [68] should be avoided.

The concept of combining a broad-spectrum antibiotic with a substance with known efficacy against *C. difficile* to prevent CDI can currently not be supported. There has been a study examining the addition of intravenous metronidazole without benefit concerning CDI rates [69]. A number of studies seem to confirm the efficacy of oral vancomycin as secondary prophylaxis for patients at risk for recurring CDI [70, 71]. Studies examining the prophylactic addition of vancomycin to an antibiotic regimen as primary prophylaxis are currently in progress (e.g., ClinicalTrials.gov NCT02951702). Unfortunately, there are as yet no reliable data concerning critically ill patients in the ICU.

One of the most challenging situations is presented if a patient has already had recurrent or refractory CDI but it is not possible to terminate antibiotic therapy. Particularly, coinfections with multidrug-resistant pathogens like vancomycin-resistant *Enterococcus faecium* are increasingly common and challenging. In most cases it is not possible to find an antibiotic with equally good performance against *C. difficile* and the multidrug-resistant pathogen. We would recommend choosing the best antibiotic for the coinfecting pathogen and combining with vancomycin to prevent recurrence of CDIF. Colonization with multidrug-resistant pathogens may be a reason to consider early FMT, which has been reported to reduce the carriage of resistant bacteria.

### Transmission in the ICU

With all of these factors considered and probably even a risk score employed, the next issue should be to reduce the rate of CDI in high-risk patients. A key point to contemplate is where and how the patient acquires the pathogenic *C. difficile* strain. This is complicated by the fact that acquiring the strain and developing symptomatic disease may be distinct events. Nevertheless, preventing transmission is bound to decrease the rates of symptomatic infection. Recent studies using whole genome sequencing were able to demonstrate that the primary mode of transmission is not from patient to patient but by contact with remaining spores in the environment or on the hands of healthcare personnel [72]. As a graphic example, Gerba et al. [73] were able to cultivate *C. difficile* from ICU touch screens. Considering the number of colonized and asymptomatic patients, it may be necessary to discuss performing *C. difficile* decontamination in the rooms of asymptomatic carriers, implying a screening of all patients for preventive purposes. Increased hygienic measures (regular disinfection of high-risk surfaces, hand hygiene) should be put in place for high-risk patients. In accordance with the proposed mode of transmission via the environment, single-room treatment of patients is not proven to significantly decrease the CDI rate [74]. Not even the beneficial effect of isolating symptomatic patients is clearly established.

Symptomatic patients with a high clinical suspicion of CDI should receive stool testing as soon as possible. Until the results are available, those patients have to be treated as CDI-positive to prevent further transmission within the ICU.

### Other approaches

One of the most controversial topics concerning prevention is the use of probiotics. Advocates hope for nonantibiotic ways to prevent, cure, and avoid recurrence of CDI. Concerning prevention, there is a multitude of studies with various end results. The latest Cochrane meta-analysis [75] shows certain probiotics to be effective for preventing CDI. Unfortunately, the variety of different strains, dosages, indications, and treatment durations severely impairs interstudy comparability and results in medium-quality data. The conclusions of the report are explicitly limited to patients who are not “immunocompromised or severely debilitated”. Taking into account the discussion around possible harmful effects of probiotic for ICU patients triggered by the negative results of the PROPATRIA trial for patients with pancreatitis, we would hesitate to generally recommend probiotics for the prevention of CDI in the ICU based on the current data.

Results from a current phase III study (CDiffense) on a vaccine based on Clostridium spore proteins [76] are expected this year. Other groups have begun to examine the effects of a purposeful colonization of the colon with nonpathogenic strains of *C. difficile* [77]. Iron-saturated bovine lactoferrin has been shown to delay *C. difficile* growth and toxin production in vitro [78]. These potential new tools in our preventative armamentarium are still far from routine clinical use, and none have been evaluated in the ICU setting.

## Conclusions

- About 10% of patients with diarrhea will test positive for CDI. Around 2% of ICU patients develop an episode of CDI.
- Estimating the severity of CDI is essential for prognosis and therapy. Diagnosis and estimation of disease severity and progression are even more complicated in the ICU setting and should be assisted by clinical prediction tools (i.e., ATLAS score). Current diagnostic algorithms may lead to an underestimation of CDI severity in ICU patients.
- Testing should include direct toxin testing by ELISA. We do not consider the isolated detection of *C. difficile* via PCR sufficient to make the diagnosis of CDI.
- 10 to 20% of patients show an asymptomatic colonization with *C. difficile* without disease symptoms. The prognostic consequence for the asymptomatic carrier is not clear.
- Oral and, if needed, topical application of vancomycin is still the backbone of antibiotic treatment.
- Early recognition of treatment failure is still an unresolved clinical problem. In the case of treatment failure, alternative treatments include substituting vancomycin with fidaxomycin, tigecycline, a combination of intravenous metronidazole and vancomycin, immunoglobulins, and FMT.
- Preventative measures and an acute awareness of risk factors should be a priority in every ICU. The clinical team should be aware of the individual risk profile of each patient for developing CDI while in the ICU. Where possible, this risk should be minimized using a set of preventive bundles. These should include involving an ID specialist and reducing or terminating antibiotic therapy, discontinuation or replacement of PPI therapy, and increased and predefined hygienic measures

## Abbreviations

ABS: Antibiotic stewardship
CDI: *Clostridium difficile* infection
ELISA: Enzyme-linked immunosorbent assay
FMT: Fecal microbiota transplantation
GDH: Glutamate dehydrogenase
ICU: Intensive care unit
IDSA: Infections Diseases Society of America
NAAT: Nucleic acid amplification test
PCR: Polymerase chain reaction
POCT: Point of care testing
PPI: Proton pump inhibitors
ULN: Upper limit of normal
